# Carbon Electrodes in Perovskite Photovoltaics

**DOI:** 10.3390/ma14205989

**Published:** 2021-10-12

**Authors:** Preawpun Pradid, Kanyanee Sanglee, Non Thongprong, Surawut Chuangchote

**Affiliations:** 1 Nanoscience and Nanotechnology Graduate Program, King Mongkut’s University of Thonburi (KMUTT), 126 Prachauthit Road, Bangmod, Thungkru, Bangkok 10140, Thailand; pr.preawpun@gmail.com (P.P.); non.tho@kmutt.ac.th (N.T.); 2 Solar Photovoltaic Research Team (SPVT), National Energy Technology Center (ENTEC), National Science and Technology Development Agency (NSTDA), 114 Thailand Science Park, Phahonyothin Road, Khlong Luang 12120, Thailand; kanyanee.san@entec.or.th; 3 Department of Tool and Materials Engineering, Faculty of Engineering, King Mongkut’s University of Technology Thonburi (KMUTT), 126 Prachauthit Road, Bangmod, Thungkru, Bangkok 10140, Thailand; 4 Research Center of Advanced Materials for Energy and Environmental Technology (MEET), King Mongkut’s University of Technology Thonburi (KMUTT), 126 Prachauthit Road, Bangmod, Bangkok 10140, Thailand

**Keywords:** carbon, electrode, perovskite solar cell, printing

## Abstract

High-performance lab-scale perovskite solar cells often have a precious metal as the top electrode. However, there are drawbacks to using metal top electrodes on a large scale, such as inducing degradation processes, requiring a high-temperature deposition process under vacuum, and having low scalability. Recently many studies have shown the potentials of using a carbon electrode because of its conductivity, flexibility, low cost, and ease of fabrication. This review article presents an overview of using carbon materials to replace the top electrode in perovskite photovoltaics. We discuss various fabrication techniques, various carbon-based device structures, and the advantages of using carbon materials. A collection of research works on device performance, large-scale fabrication, and device stability is presented. As a result, this review offers insight into the future of large-scale flexible solar cells.

## 1. Introduction

Using solar cells to convert sunlight into electricity provides various benefits, including sustainability, reducing pollution, and being environmentally friendly. However, compared with other types of renewable energy, energy production using silicon solar cells, which are the most commonly available nowadays, is still relatively expensive. As a result, photovoltaic research for less expensive solar energy is currently ongoing. Recently, perovskite solar cells (PSCs) have emerged as the next generation of solar cells because of their high energy conversion efficiency and low production cost [1].

Perovskite crystals can be entirely inorganic or hybrid organic-inorganic compounds. The chemical formula for perovskite is ABX_3_; where the A site contains a cation, such as formamidinium ion (HC(CH_2_)_2_^+^ or FA), methylammonium ion (CH_3_NH_3_^+^ or MA), cesium ion (Cs^+^), and their mixture; the B site contains a divalent metal cation (e.g., Pb^2+^, Sn^2+^); and the X site is a halide anion (e.g., I^−^, Br^−^, Cl^−^) [2]. Some outstanding properties of perovskite include high light absorption coefficient, tunable bandgaps through compositional control, and low exciton binding energy [3].

In under a decade, the power conversion efficiency (PCE) of perovskite solar cells has risen from a single digit to over 20%. The photovoltaic function of perovskite was first discovered in a liquid electrolyte sensitizer photovoltaic cell containing CH_3_NH_3_PbI_3_ nanocrystalline sensitizer on TiO_2_ mesoporous substrate with the PCE of 3.8% in 2009 [4]. In 2013, Henry J. Snaith reported the emergence of new solid-phase perovskite solar cell structures that marks the new era for low-cost, high-efficiency solar cells [5]. To date, the highest certified PCE of perovskite solar cells is at 25.5%, according to the National Renewable Energy Laboratory (NREL) chart [6]. From the chart, the efficiency of perovskite solar cells is still in an upward trend, which has piqued the interest of both the research community and the business sector.

**Figure 1:**
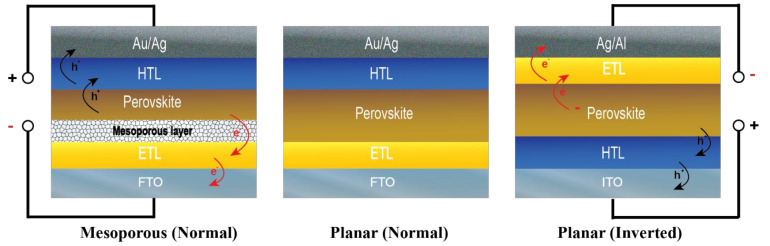
Types of perovskite solar cells, mesoporous and planar structures.

Typical planar perovskite solar cell structures consist of five layers, which are a perovskite light-absorbing layer, an electron transporting layer (ETL), a hole transporting layer (HTL), cathode, and anode electrodes, as shown in Figure 1. The bottom electrode is usually a glass or plastic substrate coated with transparent conducting oxide (TCO). The ETL is a metal oxide semiconductor such as titanium dioxide (TiO_2_), tin dioxide (SnO_2_), or zinc oxide (ZnO) [7,8,9]. Poly[bis(4-phenyl)(2,4,6-trimethylphenyl)amine] (PTAA), copper thiocyanate (CuSCN), 2,2′,7,7′-Tetrakis[N,N-di(4-methoxyphenyl)amino]-9,9’-spirobifluorene (spiro-OMeTAD) are common HTL materials [10,11]. Metallic electrodes, such as gold (Au), silver (Ag), and aluminum (Al), are used for the top electrode and are prepared using a thermal evaporation process. 

However, because of the high cost of precious metals and their interaction with the HTL layer that speeds up perovskite solar cell degradation [12], utilizing Au and Ag for the top electrode may not be the ideal choice for large-scale perovskite solar cell production. For Al, its electrodes are used in only planar (p–i–n) structures (Figure 1). As a result, one of the primary goals of perovskite solar cell research is to find alternatively low-cost materials for the top electrode, which can be used in several structures of perovskite solar cells.

Unique mechanical and electrical properties of carbon materials have piqued researchers’ interest in many fields, including supercapacitors [13], photovoltaics [14], sensors [15], conductive ink [16,17], lithium-ion batteries [18], and redox flow batteries [19]. Carbon nanotube ink’s potential, according to Ryan P. Tortorich et al., includes flexible and transparent printable electrodes due to low surface tension and the ability to be printed on various substrates, including paper, glass, and flexible substrates [20]. These carbon applications coincide with the advancements of perovskite solar cells, making carbon materials promising field additives.

Replacing the metallic electrode with carbon-based materials has gained much attention recently, owing to their chemical stability, being abundant, low preparation cost, good electrical conductivity, and hydrophobic properties [21,22]. Several techniques, such as screen printing, doctor blade, and spray, can be used to make a conductive electrode from solution-based carbon, and they offer more advantages over high-temperature thermal evaporation approaches. A carbon material with a work function of 5.0 eV has been demonstrated to be a suitable replacement for the Au electrode, with a work function of 5.1 eV [23]. Commercial carbon materials in a variety of forms, including carbon paste, carbon black, carbon ink, graphite, graphene, and others, have been successfully applied to perovskite solar cells [24,25,26]. According to a recent article [27], an ecologically friendly perovskite solar cell using bio-carbon electrodes prepared by carbonizing biomass can have a PCE as high as 12.82 percent. Using carbon materials for the back contact is also promising for flexible photovoltaics and wearable electronic applications [28,29].

In this review, the use of carbon-composite materials in perovskite solar cells, focusing on electrode applications, is discussed. In Section 2, the structures of carbon electrode-based devices, as well as their benefits for increasing efficiency, long-term stability, and lowering environmental impact, are reviewed. Section 3 summarizes various carbon fabrication techniques, as well as their corresponding device structures and performance. Section 4 summarizes large-scale perovskite solar cell developments, especially in the stability and efficiency perspectives. Finally, in Section 5, this review is concluded, and some outlooks on the future of perovskite photovoltaics with carbon layers are provided.

## 2. Features of Carbon Electrodes in Perovskite Photovoltaics

### 2.1. Structure of Carbon Electrode-Based Perovskite Photovoltaics

Carbon can be found in natural allotropes (such as diamond and graphite) as well as other synthesized forms such as fullerenes, carbon nanotubes, graphene, and carbon black [30]. Two-dimensional carbon allotropes can have an extended network of sp^2^ hybridized structures like in Graphite [31] or a very large polyaromatic hydrocarbon [32]. Graphite absorbs a broad spectrum of wavelengths, from deep ultraviolet to radio frequencies [33]. For diamond, the sp^3^ tetrahedral structure results in hardness, low electrical conductivity, and optical transparency in visible wavelengths. Carbon nanotubes (CNTs) are one-dimensional rolled-up graphene sheets with high thermal and electrical conductivity, low thermal expansion, and high flexibility [34]. 

Carbon materials have properties suitable for use as electrodes in perovskite solar cells, such as electron transfer surface coverage, absorption, and conductivity. Carbon paste made from carbon composites and conducting polymers can be used to fabricate carbon electrodes. This approach is more practical and cost-effective than metal electrode deposition under vacuum using an e-beam evaporator [35] or thermal evaporation [36], both of which have limited scalability. Huiyin Zhang et al. compared perovskite solar cells with the structure of FTO/TiO_2_/perovskite/Spiro-OMeTAD/electrode but different top electrodes [37]. The results showed that carbon electrodes prepared by doctor blade techniques result in PCE values as high as 19.2%, comparable to the devices with evaporated Au electrodes with a PCE of 20%. In addition, the device made of carbon electrodes has significantly improved long-term stability. After 1000 h of storage in an ambient atmosphere, it retained almost 95% of its initial efficiency (where the one made of Au electrodes showed the PCE of 84% of its initial efficiency). Furthermore, after 80 h aging at maximum power point under illumination and in a nitrogen atmosphere, the Au-made one faster degraded (retained around only 22% of its initial efficiency) than carbon-based cells, which could retain about 94% of its initial performance [37]. In comparison with vacuum evaporated metal electrodes, the macroporous carbon electrode has excellent interface contact, high efficiency, and better stability. Carbon materials have also been applied to supercapacitors, batteries, and electrodes because of the higher surface area with lower matrix resistivity at an acceptable cost [24,25,26].

Replacing the top metal electrode with carbon using fabrication techniques, such as dip coating, doctor blade, spray, screen printing, and inkjet printing, can result in reasonable photovoltaic performances. For example, in a work by Mario Alejandro Mejia Escobar et al. [6] hybrid perovskite solar cells with a structure of FTO/c-TiO_2_ (20 nm)/n-ZrO_2_ (25 nm)/Perovskite/Spiro-OMeTAD/Ag (120 nm) show PCE values up to 16.13%. This work used thermal evaporation for the top silver electrode and spin coating for the other layers. Compared with the work of Camellia Raminafshar et al. [38], devices with a similar structure (FTO/mp-TiO_2_/mp-ZrO_2_/Perovskite/Carbon) show device efficiencies of up to 9.5% in ambient conditions with 20–30% humidity and no encapsulation. One benefit of the carbon electrode, according to the research, is its hydrophobicity. However, the study of layer thickness effects in this work suggested optimal thicknesses of the carbon electrode, mp-TiO_2_, and mp-ZrO_2_ as 25 μm, 400 nm, and 1.7 μm, respectively.

Carbon nanotube-based hole transporting material and hole contact are exciting options for stable perovskite solar cells [39]. Namyoung Ahn et al. demonstrated that by combining encapsulation and carbon sandwiched perovskite solar cells (ITO/C_60_/MAPbI_3_/P3HT/CNT), the cost of the device can be reduced by 5.5% while maintaining stability for up to 2200 h under the sun [40]. Another example is the work of Kerttu Aitola et al., who reported on the HTL layer using a mixed single-walled carbon nanotube (SWCNT) and drop-cast Spiro-OMeTAD composite [41]. While devices with an Au contact have a PCE of around 18%, the SWCNT device has PCE values up to 15.0%. However, the Au-based device shows exponential degradation at an elevated temperature of 60 °C, with a 20% loss of the PCE in the first 8 h. Evidently, Au ion migration is the reason for the rapid loss of performance. Conversely, the SWCNT device demonstrated a slow decay, estimated using a linear function with a -0.005% h-1 slope, assuming this trend continues.

### 2.2. Benefits of Carbon Electrodes in Perovskite Photovoltaics

Aside from the practicality and low cost of carbon materials, there are many other advantages to using them in perovskite solar cells. Using different forms of carbon as additives to each layer offers different benefits, as summarized in the review article by Ferguson et al. [42]. Quantum dots made of carbon and graphene, for example, improve the thermal stability of transporting layers. Carbon nanotubes improve electrical conductivity significantly. Further, the mechanical stability of free-standing graphene could be the future of flexible perovskite solar cells. 

Graphene’s high electrical conductivity and light transmittance make it an excellent material for flexible electrodes. Peng You et al. demonstrate the fabrication of graphene electrodes in semitransparent perovskite solar cells using a chemical vapor deposition (CVD) method, resulting in PEDOT-doped two-layer graphene: PSS layer with a sheet resistance of 140 ± 35 sq^−1^ and visible transmittance of over 90%. Adding a stack of 1 to 4 layers of graphene electrodes causes the sheet resistance and transmittance to drop. By optimizing the number of graphene layers, they found that devices with two layers of graphene bring about the highest PCE of 12.02% ± 0.32%. However, because of imperfections in the graphene layers and increased surface roughness, efficiencies of devices with 3 or 4 layers of graphene electrodes decrease. This research suggests that CVD graphene is an excellent candidate for flexible and transparent electrodes in perovskite solar cells. Furthermore, the free-standing, 2D form of graphene has also proven to be an intriguing route for flexible transparent electrodes in perovskite solar cell research. A single layer of the material is stable in air and has ~97% visible light transmission, which offers great potential for further development [43]. Another critical benefit of graphene is that solution-based graphene can be used in scalable processes for low-cost printable electronics [44].

Habisreutinger et al. reported charge transfer properties of carbon nanotubes (CNTs) in extracting photo-generated holes in perovskite devices [34]. According to the paper, CNTs can perform a variety of purposes, including additive and interface modifiers, as well as hole-transporting systems and charge-selective layers. CNT inherent robustness, stability, outstanding charge transport properties, and intrinsic mechanical flexibility appear to be significant advantages over other materials. CNT-based devices could become a highly preferable option for high-performance, long-term stable devices if their PCEs improve further. CNT morphology can also provide advantages in structural properties and high porosity, which have already been shown to be key factors in supercapacitors [26]. A.G. Pandolfo reported that a variety of porous forms of carbon are currently more favorable than metal electrodes because of their extremely high surface areas, relatively high electronic conductivity, and acceptable costs [26].

**Figure 2:**
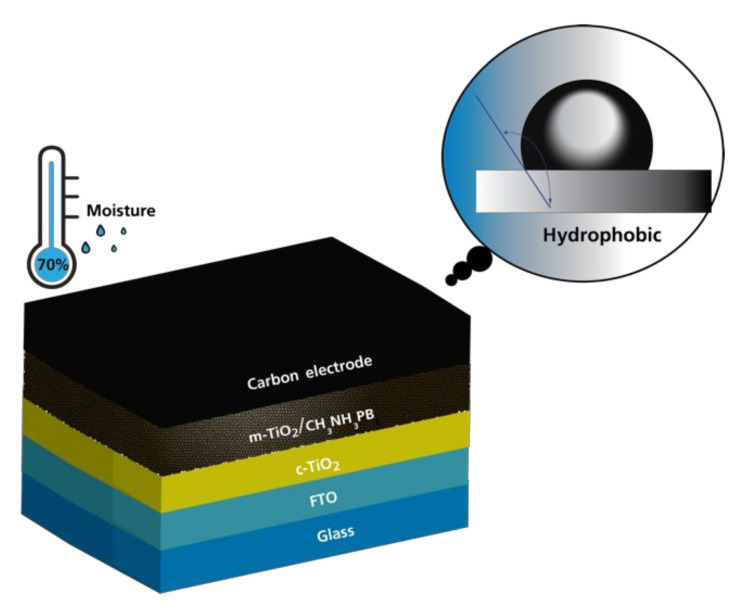
Schematic illustration of the perovskite solar cells and contact angel of H_2_O on carbon. Modified after Huawei Zhou et al., (2014) [46].

Combining different allotropes of carbon to maximize device performance and stability is also a fascinating route, as suggested by Rui He et al. [22] Carbon materials’ excellent properties could improve perovskite solar cell lifetime, stability, and performance when multiple different layers of carbon are applied. Hydrophobic 0D to 3D carbon materials can also protect the perovskite layer from moisture and degradation caused by high temperatures. At high operating temperatures, PCE loss can be attributed to perovskite deterioration. High moisture levels are still an issue because water molecules can be absorbed by perovskite precursor materials, changing their properties, causing poor stability and degradation. The water-soluble MA iodide degrades the perovskite crystal structure, resulting in a lower PCE after outdoor exposure. Because of superior water resistance or hydrophobicity of carbon, carbon material can improve stability in high humidity and high temperature (Figure 2). In this sense, Wei et al. compared perovskite solar cells with Au electrodes to carbon electrodes and found that carbon film can offer good flexibility and conductivity, while thicker carbon film can cover and protect CH_3_NH_3_PbI_3_ film from high humidity [45].

## 3. Fabrication Methods for Carbon Electrodes in Perovskite Photovoltaics and Their Performances

**Figure 3:**
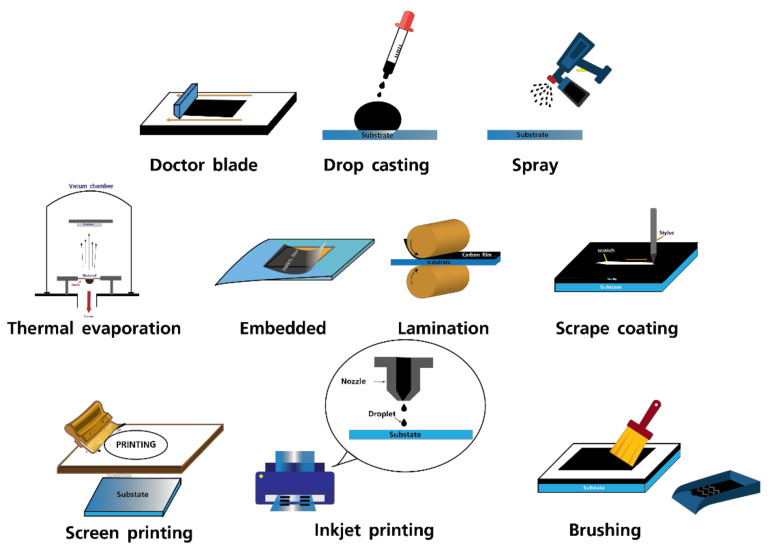
Schematic illustration of several techniques for fabrications of carbon electrodes in perovskite solar cells.

In this section, different carbon electrode fabrication techniques in perovskite solar cells (Figure 3), as well as different types of carbon materials, device structures, and efficiency parameters, have been reviewed. Table 1 summarizes the findings from the literature.

Different types of carbon and fabrication techniques produce different levels of device performance, as shown in Table 1, but carbon nanotube films from drop-casting appear to have the highest PCE with an *n–i–p* mesoporous structure, at 17.58% [62]. Multi-walled carbon nanotubes have high electrical conductivity and, due to their hydrophobicity, form a good interface with the CuSCN layer, resulting in improved perovskite solar cell stability. The well-forming interface between CuSCN and CNTs at the contact also results in a flat and smooth interface between the perovskite and CuSCN. Although the drop-casting method is simple to use, customizing the thickness can be difficult. Inkjet printing techniques allow us to design the carbon electrode more precisely and controllably [65]. However, the contact between the perovskite layer and carbon is not very well-formed, as evidenced by large pinholes being visible at the interface. Chemically engineering carbon ink is one way to solve the problem. According to the findings, adding CH_3_NH_3_CI to carbon ink can boost efficiency from 8.51%to 11.60% [65].

Il Jeon et al. [57] showed flexible inverted perovskite solar cells with transparent carbon electrodes made of single-walled carbon nanotubes (SWNTs) or graphene at the bottom as well as a comparison of ITO glass and polyethylene naphthalate (PEN) substrates. It can be seen that the structure ITO/graphene/MoO_3_(2 nm)/PEDOT:PSS/perovskite/C_60_/ BCP/Al/LiFgraphene had a PCE of 14.20% compared with 12.80% for single-walled carbon nanotubes (SWNTs). When fabricated on PEN, the PCE of graphene-based and SWSNT-based solar cells were 13.30% and 11.00% respectively. It can be seen that the PCE of graphene is higher than that of the SWNTs because the graphene film has a higher transmittance and flatter surface. It can be seen that the PCE of graphene is higher than that of the SWNTs because the graphene film has a higher transmittance and flatter surface. Atomic force microscopy (AFM) demonstrating that the average roughness value of the SWNT films was much higher (*R*_a_ = 15.1 nm) than that of the graphene films (*R*_a_ = 3.5 nm). It can be seen that graphene has a smoother surface than SWNTs. The higher *J*_SC_ is due to the intrinsically high transmittance of single-layered graphene compared with SWTN-based perovskite solar cells.

## 4. Substrates, Stabilities, and Performances of Large-Area Perovskite Photovoltaics Made of Carbon Electrodes

**Figure 4:**
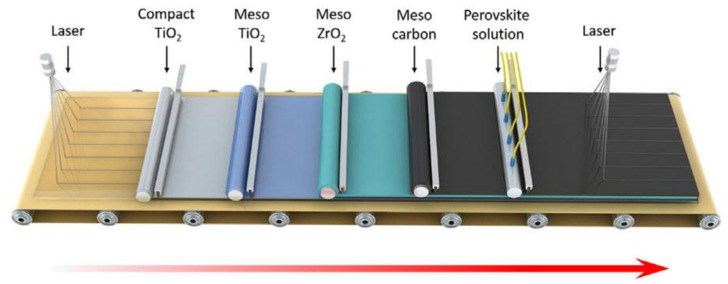
Schematic illustration of the proposed production line of large-scaled perovskite solar cells. Reprinted from Yue Hu et al., (2017), [67] Copyright (2017), with permission from WILEY-VCH Verlag GmbH & Co. KGaA, Weinheim.

We commonly know that highly efficient perovskite solar cells are achieved in the laboratory with small active areas (~0.05–1.0 cm^2^) so up-scaling film fabrication to market photovoltaic panels is necessary for the next-generation photovoltaic technology. A layer-by-layer deposition is still a required method for making large-scale devices (Figure 4). However, the production of uniform films with no pinholes, matching the interface layers in the cells, and ensuring durability are difficult. For large-scale panel production, deposition of metal electrodes in a vacuum environment is also challenging. We discuss the use of carbon electrodes in perovskite solar cells with more realistic sizes in this section.

For mass production of carbon-based perovskite solar cells, printing technologies appear to be the industrial solutions. Printing techniques have several advantages, including the ability to apply to flexible materials and being low cost. However, printing a perovskite solar cell does not guarantee a high photovoltaic function on a large scale. In Table 2, the photovoltaic performances of various large-scale perovskite solar cells with different substrates and device structures in the literature are summarized. Some remarks on the information in the table as follows are given.

Carbon-based electrodes still have been potentially applied in perovskite solar cells due to their low cost and compatibility with up-scaling processes, indicating their great mass-production potential. Carbon material characteristics are also responsible for perovskite layer protection. In comparison to metal materials, carbon is hydrophobic and does not corrode in high humidity. 

Carbon-based perovskite solar cells have promisingly low fabrication costs. For example, colloidal graphite carbon paste (PELCO^©^) was used in the research of Sawanta S. Mali et al., [70] which is available in the market for about 14.50 USD/25 g (estimated from the commercial price on the website). It can be seen that the price of carbon-based electrodes is 3 times lower compared with the prices of metal-based electrodes.

In the viewpoint of substrates of perovskite solar cells, large-scaled cells are conventionally produced on transparent conducting oxide (TCO)-coated glasses. The conductive glass material is heavy and has no flexibility, causing its ease to break. Anyway, now solar cells have a variety of applications on various material substrates. For example, flexible plastics or even papers (bendable materials) can be used as alternative substrates. It can be seen that flexible materials have begun to be used in the production of perovskite solar cells to alleviate weight issues, be easy to produce, reduce the problem of breakage when we produce solar cells in large-scale materials, and be able to use in a variety of tasks.

**Figure 5:**
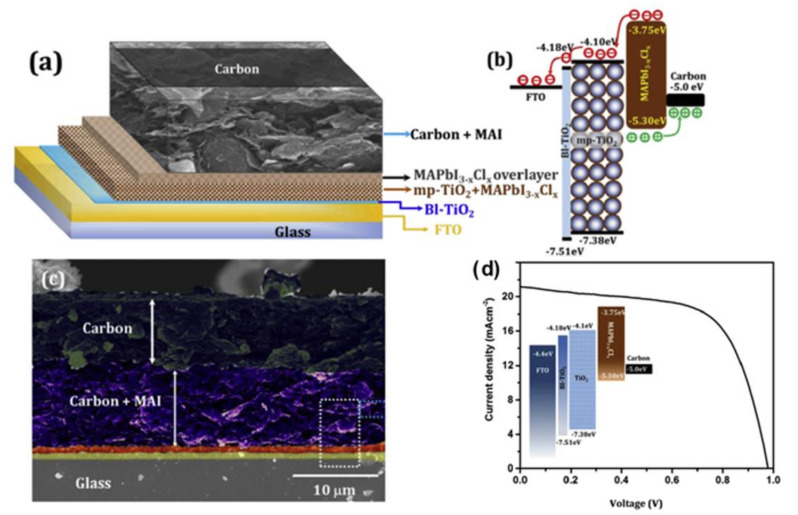
(**a**) A schematic image of the device configuration of a perovskite solar cell made of carbon electrode on a glass substrate. (**b–d**) Energy level diagram, a cross-sectional SEM image, and J-V characteristic curve of the device, respectively. Modified and reprinted from Sawanta S. Mali et al., (2017), [70] Copyright (2017), with permission from Elsevier.

In terms of stability, perovskite solar cells made of carbon electrodes on glass substrates were stable for more than 160 days in the test at room temperature under an open atmosphere with a PCE of 13.4% (Figure 5) [70]. The carbon layer was shown to be beneficial in terms of waterproofing and air stability. Table 2 also shows whether perovskite solar cells made of carbon electrodes on flexible materials or papers have moisture resistance.

**Figure 6:**
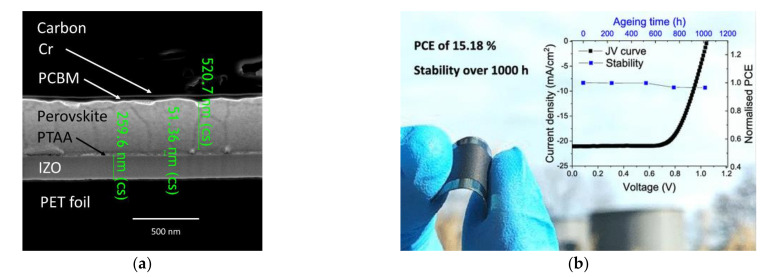
(**a**) A cross-section SEM image of a carbon electrode-based perovskite solar cell on a PET foil. (**b**) A photograph image of the carbon electrode-based flexible perovskite solar cell with (i) a photocatalytic current density and voltage curve and (ii) thermal (85 °C) stability of the device over 1000 h of aging. Reprinted from Vivek Babu et al., (2020), [74] Copyright (2020), with permission from The American Chemical Society (further permissions related to the material excerpted should be directed to The American Chemical Society).

In terms of performance, it can be seen that device on flexible material such as PET foil has a PCE of up to 15.18% (Figure 6) [74].

**Figure 7:**
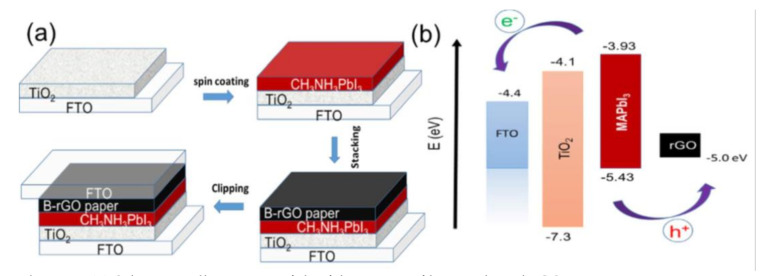
(**a**) Schematic illustration of the fabrication of boron-doped rGO paper as an HTL in perovskite solar cells. (**b**) Energy level diagram of the devices. Reprinted from D. Selvakumar et al., (2018), [28] Copyright (2018), with permission from Elsevier.

Duraisamy Selvakumar et al. [28] demonstrated that incorporation of boron into reduced graphene oxide (B-rGO) to be used for the hole transport material (HTM) of the devices on FTO paper could improve conductivity and mobility (Figure 7). They found that their FTO/TiO_2_/CH_3_NH_3_/PbI_3_/B-rGO/FTO sandwich structure with an active area of 1 cm^2^ performed better for flexible solar devices, showing the highest PCE value of 8.96%.

Yue Hu et al. demonstrated large-area perovskite solar cell fabrication on FTO glass substrates, which could be used in industrial applications [67]. They used screen printing to deposit all the layers, including the top electrode, which is made of graphite and carbon. According to the paper, the thickness alignment of the triple mesoscopic layer was optimized to achieve a PCE of 14.02% for a single cell under illumination intensity of 100 mW/cm^2^. They further developed a 10 serially connected cells module (10 × 10 cm^2^) with an active area of 49 cm^2^ that shows a PCE of 10.4%. They also conducted multiple stability tests, including 1000 h under light soaking, one month in an outdoor setting, and over a year of shelf-life stability.

Amba Bashir et al. developed large-scale carbon-based perovskite solar cells by incorporating screen-printed carbon and a p-type inorganic interlayer of spinel cobaltite oxides (Co_3_O_4_) [69]. The carbon and Co_3_O_4_ layers serve the purposes of repelling moisture and suppressing charge recombination, respectively. These approaches improve energy level matching, efficiency, and stability, resulting in an efficiency of 13.27%, up from 11.25% in a standard carbon-based perovskite solar cell, and a cell that is stable for 2500 h under ambient conditions. They also created a monolithic perovskite module with a 70 cm^2^ active area and a PCE of over 11%.

Using carbon as the HTL was also shown by Sawanta S. Mali et al. [70]. They modified commercially available carbon ink with methylammonium lead iodide (MAI) and used a doctor blade technique to fabricate perovskite solar cells having a device configuration Glass/FTO/mp-TiO_2_/MAPbI_3-x_Cl_x_/carbon + MAI/Carbon. An optimized device can sustain the PCE for 160 days in ambient air and showed a PCE of 13.04% for a large area device (1.1 cm^2^).

Many studies, according to Table 2, used carbon electrodes in an HTL-free perovskite solar cell configuration. Zhiyong Liu et al., for example, used a modified two-step approach for CH_3_NH_3_PbI_3_ nanocrystalline deposition under high humidity and a printed carbon counter electrode to create HTL-free devices without encapsulation [71]. The device exhibited superior stability in the air in the dark (temperature ~20 °C, humidity ~20%) with a PCE of 6.21% for over 2000 h.

## 5. Conclusions and Outlook 

In order to overcome the limitations of metal electrodes used in perovskite solar cells, such as high-temperature and vacuum processes, humidity sensitivity, poor scalability, and price, carbon is a promising alternative. Carbon materials have many advantages over metal electrodes, including eco-friendliness, flexibility, hydrophobicity, high surface area, and porosity. Because carbon is hydrophobic, using it as the HTL and the top electrode can protect the device from moisture, resulting in an air-stable solar cell even without encapsulation. These properties pave the way for a wide range of future applications, including flexible air-stable solar panels that can be attached to a variety of surfaces at a lower cost. Wearable electronics and indoor photovoltaics are also catching on in terms of technological advancement. Carbon materials are also appealing commercially because of their lower cost and impressive performance.

Future carbon materials research for perovskite solar cells may include synthesizing less expensive carbon materials and investigating the potential of carbon nanoparticles. Typically, powdered carbon is used to create a solution prior to use in many research works. We can change the properties of the resulting film by synthesizing nanoparticles of various sizes and types as powders or as precursor solutions. 

In the present review paper, we discuss the advantages of replacing metal electrodes and HTL materials with carbon materials. We go over various carbon-film fabrication techniques as well as device structures. The advantages of using carbon materials, such as superior optical, electrical, and mechanical properties, have positive effects on the perovskite solar cell lifetime, stability, and performance. We can achieve long-term stability, good flexibility, and high performance in carbon-based perovskite solar cells, according to the research we compiled in this work. Another advantage of carbon is the availability of a variety of low-temperature, low-cost, large-scale fabrication techniques. Several studies have successfully demonstrated how to fabricate a large-scale flexible substrate from carbon materials using inkjet printing, screen printing, and roll-to-roll printing.

## Figures and Tables

**Table 1:** Summary of several techniques for fabrications of carbon electrodes in perovskite solar cells.

Technique	Type of Carbon	Device Structure	Jsc (mA/cm^2^)	Voc (V)	FF	PCE (%)	Ref.
Doctor blade coating	CuPc-doped Carbon	FTO/TiO_2_/Perovskite/ Carbon	21.40	1.02	0.68	14.80	[47]
	Carbon paste	FTO/SnO_2_/Perovskite/ Spiro-OMeTAD/Al foil/ Carbon	19.72	1.09	0.70	15.38	[8]
		FTO/TiO_2_/Perovskite/ Carbon	22.15	0.87	0.67	13.03	[9]
		ITO/SnO_2_/Perovskite/ Carbon	22.19	1.08	0.57	13.64	[48]
		FTO/SnO_2_/Perovskite/CuSCN/ Carbon	20.40	1.08	0.66	14.60	[49]
		FTO/TiO_2_/SiO_2_/Perovskite/ Carbon	20.30	0.89	0.65	11.9	[50]
	Carbon black and graphite	FTO/TiO_2_/Al_2_O_3_/NiO/Perovskite/ Carbon	21.62	0.92	0.76	15.03	[51]
		FTO/TiO_2_/Perovskite/ Carbon	20.25	1.05	0.63	13.50	[52]
Drop casting	MWNTs	FTO/TiO_2_/Perovskite/ CuSCN/Carbon	23.70	1.01	0.73	17.58	[53]
		FTO/ c-TiO_2_/ m-TiO_2_/ Perovskite/Carbon	19.31	0.88	0.71	12.08	[54]
Spray coating	O-MWNTs	FTO/SnO_2_/Perovskite/ O-MWNTs/Carbon	21.96	0.99	0.41	8.99	[55]
Thermal evaporation	B-MWNTs	FTO/c-TiO_2_/m-TiO_2_/ Perovskite/B-MWNTs	21.35	0.90	0.76	14.60	[56]
	Al_2_O_3_-B-MWNTs	FTO/ TiO_2_/Al_2_O_3_-B-MWNTs-Perovskite	21.50	0.92	0.77	15.23	[56]
	Graphene	ITO/graphene/MoO_3_ (2 nm)/ PEDOT:PSS/Perovskite/ C_60_/BCP/Al/LiF	21.20	0.96	0.70	1420	[57]
		PEN/graphene/MoO_3_ (2 nm)/ PEDOT:PSS/Perovskite/ C_60_/BCP/Al/LiF	20.00	0.97	0.69	13.30	[57]
	SWNTs	ITO/SWNT/MoO_3_ (2 nm)/ PEDOT:PSS/Perovskite/ C_60_/BCP/Al/LiF	17.50	0.96	0.76	12.80	[57]
		PEN/SWNT/MoO_3_ (2 nm)/ PEDOT:PSS/Perovskite/ C_60_/BCP/Al/LiF	18.80	0.90	0.65	11.00	[57]
Embedded	SWNTs	SWNT-PI/MoO_x_/ poly(triarylamine) (PTAA)/ Perovskite/C_60_/BCP/Cu	19.00	1.05	0.76	15.20	[58]
Laminated	TFMS-doped SWNTs	ITO/PEDOT:PSS/Perovskite/ SWNT/Spiro-MeOTAD	22.70	1.12	0.73	18.80	[59]
	TFMS-doped SWNTs	ITO/SnO_2_/Perobskite/ SWNT/Spiro-MeOTAD	24.21	1.00	0.72	17.60	[60]
	Graphene/Carbon nanotube	FTO/TiO_2_/Perovskite/ Spiro-OMeTAD/ graphene/CNT	21.88	1.07	0.45	15.36	[61]
Scrape coating	Carbon ink	FTO/TiO_2_/ZrO_2_/Perovskite/ Carbon	22.38	0.87	0.50	9.89	[62]
Screen printing	Carbon paste	FTO/TiO_2_/ZrO_2_/Perovskite/ Carbon	21.40	0.88	0.57	10.70	[38]
		FTO/TiO_2_ /Perovskite (CNT drip)/Carbon	18.97	1.99	0.71	13.57	[63]
	Graphite paste	FTO/TiO_2_/ZrO_2_/Perovskite/ Graphite	22.82	0.90	0.60	12.40	[64]
	Boron dope graphite paste	FTO/TiO_2_/ZrO_2_/Perovskite/ B-Graphite	22.87	0.94	0.63	13.60	[64]
Inkjet printing	Carbon paste	FTO/TiO_2_/Perovskite/Carbon	15.00	0.90	0.63	8.51	[65]
		FTO/TiO_2_/Perovskite/ Carbon+CH_3_NH_3_I ink	17.20	0.95	0.71	11.60	[65]
Brushing	Carbon paste	FTO/TiO_2_/Perovskite/Carbon	21.27	1.04	0.65	14.38	[66]

CuPc, MWNTs, O-MWNTs, B-MWNT, SWCNTs, BCP, TFMS, and Al_2_O_3_ are copper phthalocyanine, multi-walled carbon nanotubes, oxidized muti-walled carbon nanotubes, boron doping of multi-walled carbon nanotubes, single-walled carbon nanotube, bathocuproine, trifluoromethane sulfonic acid, and aluminum oxide, respectively.

**Table 2:** Summary of several techniques to fabricate carbon electrodes in perovskite solar cells (PSC).

Substrate	PSCs Structure	Active Area (cm^2^)	Test Condition	Stability	PCE (%)	Humidity	Ref.
Glass	FTO/m-TiO_2_/ Perovskite/ Carbon	1	A 500 W xenon lamp (XIL model 05A50KS source units)	-	12.63	80%	[63]
	FTO/m-TiO_2_/ Perovskite/Carbon	1	The solar light simulator (Newport solar simulator, model number 6255, 150 W Xe lamp, AM 1.5 global filter) was calibrated to 1 sun (100 mW cm^−2^)	-	9.72	-	[66]
	FTO/ZnO/ Perovskite/ Carbon	1	Illumination of 100 mW/cm^2^ by a 450 W class AAA solar simulator equipped with an AM1.5G filter (Sol2A, Oriel Instruments)	140 d	15.1	-	[68]
	FTO/ZnO/ Perovskite/ Carbon	17.6	Illumination of 100 mW/cm^2^ by a 450 W class AAA solar simulator equipped with an AM1.5G filter (Sol2A, Oriel Instruments)	140 d	10.6	-	[68]
	FTO/TiO_2_/ZrO_2_/ Perovskite/ Carbon	49	AM1.5 illumination, without any additional UV filter and under simulated AM 1.5 100mW/cm^2^ sunlight.	1000 h	10.4	65–70%	[67]
	FTO/m-TiO_2_/ (Perovskite/ ZrO_2_/Co_3_O_4_)/ Carbon	70	AM 1.5G without any maskin	2500 h	11.39	70%	[69]
Glass	FTO/m-TiO_2_/ Perovskite_x_/ Carbon+MAI/ Carbon	>1.1	Illumination of 100 mAcm^2^, AM 1.5 illumination	160 d	13.04	-	[70]
	FTO/TiO_2_/ Perovskite/ Carbon	1	Illumination of one sun illumination	2000 h	6.21	~20%	[71]
	FTO/TiO_2_/ Perovskite/ Carbon	0.05	A solar light simulator (Newport solar simulator, model number 6255, 150 W Xe lamp, AM 1.5 global filter) was calibrated to 1 sun (100 mW cm^−2^)	-	14.58	-	[72]
	FTO/m-TiO_2_/ Perovskite/Carbon	0.05	Newport solar simulator, model number 6255, 150 W Xe lamp, AM 1.5 global filter	120 d	11.64	10–20%	[73]
Paper	FTO/ TiO_2_/ Perovskite/ B-rGO/FTO	1	The standard AM 1.5G conditions	250 h	8.96	60%	[28]

B-rGO = boron reduced graphene oxideLonghua Cai et al. [68] used a gas-pumping method to create a large-area perovskite solution film and screen-printed carbon electrodes on top of it. They make PSCs with two different active areas: 1 cm^2^ and 17.6 cm^2^, and then test them for 140 days in the open air. The small and large modules have PCEs of 10.6% and 15.1%, respectively. After the testing period, they discovered no significant degradation. Their method allows for the practical fabrication of high-performance large-area PSCs.

## Data Availability

Data sharing is not applicable.
